# Reward components of feeding behavior are preserved during mouse aging

**DOI:** 10.3389/fnagi.2014.00242

**Published:** 2014-09-16

**Authors:** Mazen R. Harb, Nuno Sousa, Joseph Zihl, Osborne F. X. Almeida

**Affiliations:** ^1^Max Planck Institute of PsychiatryMunich, Germany; ^2^Portugal and ICVS/3B's-PT Government Associate Laboratory, Institute of Life and Health Sciences (ICVS), University of MinhoBraga, Portugal; ^3^Department of Neuropsychology, Ludwig Maximilian UniversityMunich, Germany

**Keywords:** aging, associative learning, conditioning, motivation, food reward, hedonic preference

## Abstract

Eating behavior depends on associations between the sensory and energetic properties of foods. Healthful balance of these factors is a challenge for industrialized societies that have an abundance of food, food choices and food-related cues. Here, we were interested in whether appetitive conditioning changes as a function of age. Operant and pavlovian conditioning experiments (rewarding stimulus was a palatable food) in male mice (aged 3, 6, and 15 months) showed that implicit (non-declarative) memory remains intact during aging. Two other essential components of eating behavior, motivation and hedonic preference for rewarding foods, were also found not to be altered in aging mice. Specifically, hedonic responding by satiated mice to isocaloric foods of differing sensory properties (sucrose, milk) was similar in all age groups; importantly, however, this paradigm disclosed that older animals adjust their energy intake according to energetic need. Based on the assumption that the mechanisms that control feeding are conserved across species, it would appear that overeating and obesity in humans reflects a mismatch between ancient physiological mechanisms and today's cue-laden environment. The implication of the present results showing that aging does not impair the ability to learn stimulus-food associations is that the risk of overeating in response to food cues is maintained through to old age.

## Introduction

Metabolic status, a reflection of eating habits, is an important determinant of an individual's physical and mental health trajectory, especially from middle age onwards when the incidence of metabolic syndrome rises steeply (Mathus-Vliegen et al., 2012). Despite evidence linking overweight and obesity to increased risk for affective (Dixon et al., 2003; Preiss et al., 2013) and cognitive disorders (Fitzpatrick et al., 2009; Smith et al., 2011; Dahl et al., 2013; Ravona-Springer et al., 2013) and other age-related debilitating conditions (Gregor and Hotamisligil, 2011; Mathus-Vliegen et al., 2012), the neurobiology of eating behavior as a function of age remains a relatively unexplored area; most published studies focus on understanding age-related loss of appetite and body weight (Frutos et al., 2012), rather than the rising tide of obesity in older individuals (Fakhouri et al., 2012).

Feeding is a motivated behavior, driven by hunger (energy needs) but also by the reward salience of foods, represented by sensory (odor, visual appearance, taste, and texture) and physical (energy content) attributes of a given food (Mehiel and Bolles, 1988; Rolls, 2010; Beeler et al., 2012; Fernstrom et al., 2012; Desmarchelier et al., 2013; Li et al., 2013). While hunger provides the primary motivation to eat, food-seeking (wanting/liking) and ingestive behavior may be triggered by associations between the real or anticipated higher reward value of foods in a particular environmental context or because certain foods are imbued with hedonic properties (Berridge and Robinson, 2003; Berridge et al., 2009; Ferriday and Brunstrom, 2011; Ziauddeen et al., 2012). Conditioned (learned) stimuli can increase subliminal motivation to seek and consume foods during states of satiation and in excess of actual energetic demands, eventually leading to obesity.

Children and adolescents are more responsive and sensitive to external paired (conditioned) cues and the rewarding properties of palatable food than adults (Friemel et al., 2010; Birch and Anzman-Frasca, 2011; Scully et al., 2012). Normal aging is accompanied by gradual structural and functional changes in brain areas involved in sensory, reward and cognitive processing (Marschner et al., 2005; Burke and Barnes, 2006). While age-related impairments of declarative and working memory have been extensively studied (Hedden and Gabrieli, 2004; Driscoll and Sutherland, 2005), little is known about the influence of aging on non-declarative associative memory which is relevant to conditional learning (Birch and Anzman-Frasca, 2011; Petrovich, 2013). The present study examined whether implicit memory (associative learning), motivation and food preference (triggered by the food's hedonic qualities) are affected during aging in the mouse. Our results show that old mice do not suffer from impairments in their (i) ability to perceive hedonic stimuli, (ii) motivation to consume rewarding foods, and (iii) capacity to learn stimulus-food associations. Interestingly, mice also maintain their ability to adjust their calorific intake according to their metabolic status.

## Materials and methods

### Animals

Male mice (C57BL6 strain, Charles River, Sulzfeld, Germany), aged 3 (young), 6 (middle-aged) and 13–15 (old) months were used in these experiments. All procedures were carried out in compliance with national regulations on animal welfare and experimentation and European Union Directive 2010/63/EU. Animals were housed in pairs under standard laboratory conditions with *ad libitum* access to water, unless specifically mentioned. Behavioral tests (see below), each carried out in different batches of animals, were conducted during the animals' daily period of activity (diurnal phase of darkness; lights off: 7 a.m.) after 1 week of habituation to the experimental room and experimenter. In keeping with standard procedures, mice were placed on a calorie-restriction schedule to reduce body weights by 10–15% (body weights monitored daily), unless specifically stated otherwise. Animals that displayed overt signs of pathology (cf. Ladiges et al., 2013) during autopsy at the end of each experiment were excluded from final analyses; the exact number of animals used in each experiment is given in the Results Section and corresponding figure legends.

### Operant (instrumental) conditioning

Tests were performed in automated touchscreen chambers (Horner et al., 2013). The touchscreen, located opposite to the food magazine, was covered with a black Perspex mask, partitioned by three single rectangles. The conditioned stimulus (white light) was presented through the middle partition only, a tone was presented when the mouse touched the screen with its snout. The stimulus was then extinguished and a liquid reward [15 μl of diluted condensed milk (14% sugar)] was delivered into the (now illuminated) food tray. A test session comprised 20 presentations of the light stimulus-reward delivery cycle. In order to minimize between-trial interference, a variable interval (VI) schedule (10–40 s) was used. Each mouse experienced 1 conditioning session/d that was terminated when criterion was reached (completion of 20 trials in <20 min/session on at least 3 consecutive days) or after 60 min. Animals were habituated to the liquid reward and test chambers (3 d) before actual testing. The following parameters were recorded and computed during each operant conditioning session: (i) trials completed/session, (ii) time to complete session, (iii) beam breaks/min, and (iv) stimulus touches/min.

### Pavlovian (classical) conditioning

Autoshaping was performed in automated touchscreen chambers, as previously described (Horner et al., 2013). The neutral stimulus (CS) was a 10 s flash of white light in either the left- (50% of animals) or right- (50% of animals) hand side of the screen. Immediately after stimulus offset, a liquid food reward [15 μl of diluted condensed milk (14% sugar)] was delivered into the food magazine. During task acquisition mice were trained to associate the light stimulus (CS+) with reward delivery. During each session (1/day), presentations of 15 CS+ and 15 CS− were made in a randomized order (maximum of 2 consecutive presentations of same stimulus, VI schedule of 10–40 s between each stimulus). Animals reaching the criterion of 70% of correct (CS+) approach responses/session on at least 3 consecutive days were designated as sign trackers (ST) (Harb and Almeida, 2014). Mice that reached the criterion of 70% correct responses/session to the CS+ on at least 3 consecutive days were designated as ST. Those that made >80% approaches to the food (US) magazine (<20% approaches to the CS+) were categorized as Goal Trackers (GT), and those that made 20–70% approaches to the CS+ (alternated between CS+ and US with approximately equal frequency) were considered to be Intermediate Trackers (IT) (Harb and Almeida, 2014).

### Tests of motivation and hedonic preference

Two tests were used to examine motivation to retrieve a reward. The first was carried out over 2 d in the mouse touchscreen chambers; wanting/motivation was assessed by monitoring individual latencies to retrieve all of the reward and the rate of food tray entries. During each session, mice were presented with 15 liquid food rewards (15 μl condensed milk, containing 14% glucose), delivered at a variable interval (VI) 10–40 s. The second test was designed so as not to be confounded by satiety levels and energetic state. Specifically, it compared hedonic preference for one of two isocaloric drinking solutions (15% sucrose vs. milk whose fat content was 5%) presented in the home-cage. The test was conducted in a state of satiation and mice had access to their standard solid diets throughout the test. The volume of each liquid consumed was measured at 3, 6, and 24 h thereafter; the caloric intake provided by the liquid diets was computed as a function of standard chow consumption (weight and calories/body mass/d).

### Data analysis

Data were subjected to statistical analysis using Prism 5.0 statistical software package (GraphPad, La Jolla, CA). Data were subjected to either One- or Two-Way ANOVA, followed by Bonferroni post-test comparisons, or by *t*-tests, as appropriate. The minimum level of significance was set to *p* ≤ 0.05.

## Results

### Operant conditioned learning is not impaired during aging

Operant or instrumental conditioning involves learning to associate an action with an outcome; the paradigm requires that the subject “works” (here, nose-poking the light stimulus) in order to receive a reward (here, sweetened milk).

**Figure 1:**
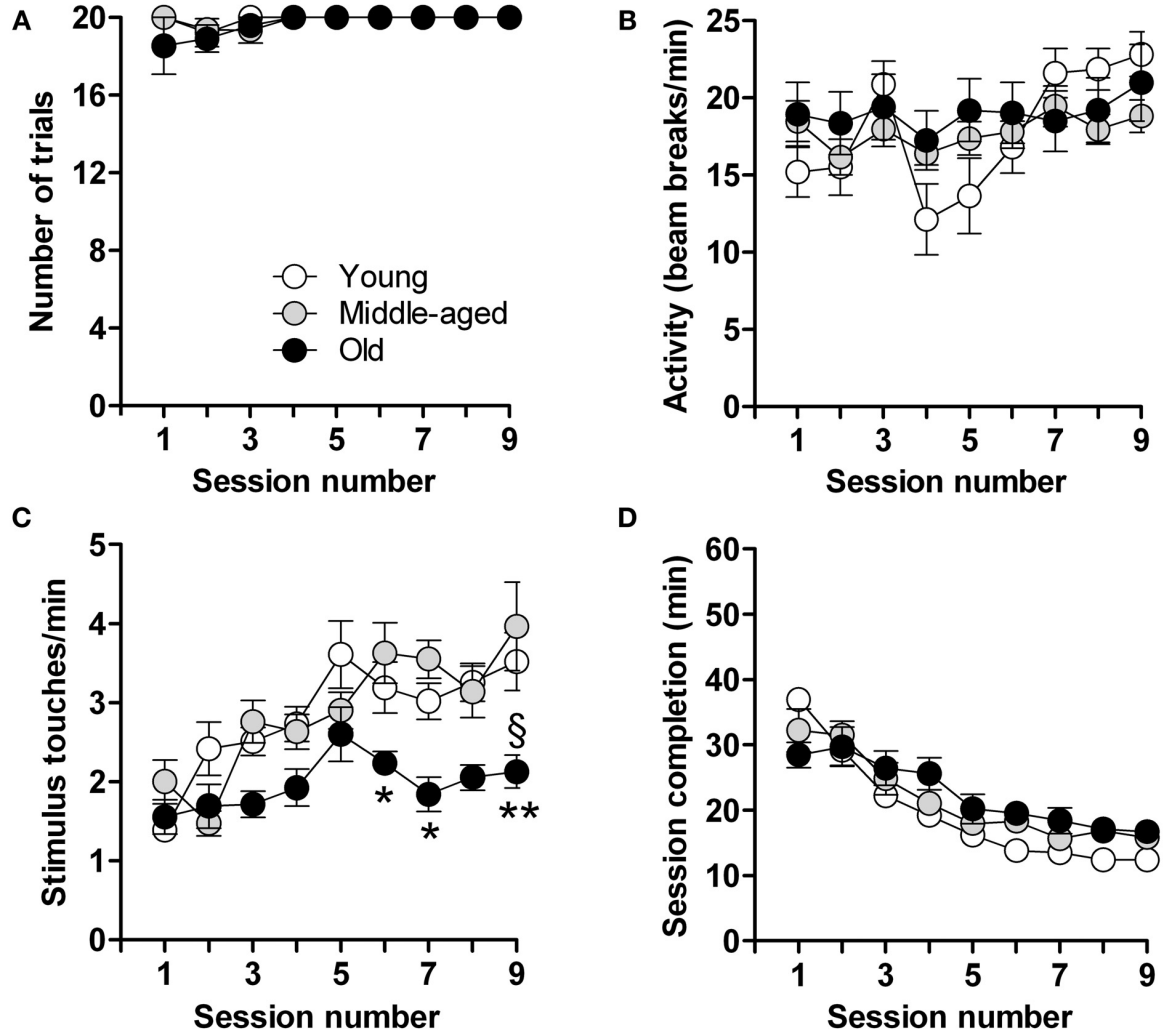
**Acquisition of operant conditioning**. Shown are the data in each of the 9 test sessions consisting of 20 trials in young (*n* = 18), middle-aged (*n* = 15) and old (*n* = 12) mice. **(A)** Number of trials completed. **(B)** Locomotor activity (infra-red beam breaks/min). **(C)** Number of stimulus touches/min. **(D)** Time (min) for completion of session. Data are presented as means ± s.e.m. § indicates a significant difference (*P* < 0.05) between the young and old groups of mice. ^*^ and ^**^ indicate significant differences (*P* < 0.05 and *P* < 0.01, respectively) between middle-aged and old mice.

Calorie-restricted male mice aged 3, 6, and 15 months, hereinafter referred to as “young,” “middle-aged,” and “old” mice, were tested after 9 days when most (young: 100%, *n* = 18; middle-aged: 94%, *n* = 15; old: 92%, *n* = 12) had reached criterion (completion of 20 trials in <20 min, on least 3 consecutive days) (Figure 1A). Locomotor activity, measured in terms of photobeam breaks in the touchscreen chambers, did not differ between young, middle-aged and aged mice; all animals habituated to the experimental set-up similarly with a gradual, but significant, increase in locomotor activity over time [*F*_(8,360)_ = 3.4; *p* = 0.0008] (Figure 1B). Mice also displayed a progressive increase in the number of stimulus-directed nose-pokes over time [*F*_(8,360)_ = 9.7; *p* < 0.0001] (Figure 1C); however, the increase was more pronounced in the young and middle-aged mice [*F*_(2,360)_ = 24.2; *p* < 0.0001]. Overall, and irrespective of age, mice showed a progressive and highly significant decrease in the time needed to complete the daily sessions [*F*_(8,361)_ = 40.1; *p* < 0.0001] (Figure 1D). These results thus show that the capacity for operant learning does not deteriorate with aging.

### Capacity for pavlovian conditioning does not change with aging

Pavlovian conditioning represents another form of associative learning. It is highly conserved but individuals vary in stimulus-reward tracking patterns (Harb and Almeida, 2014). For the assessment of pavlovian conditioning, we compared the food cue-conditioned responses (CR) of young (*n* = 31), middle-aged (*n* = 34), and old (*n* = 34) male mice. In these experiments, a flash of light served as the neutral stimulus and liquid food (sweetened milk) was used as the rewarding stimulus.

**Figure 2:**
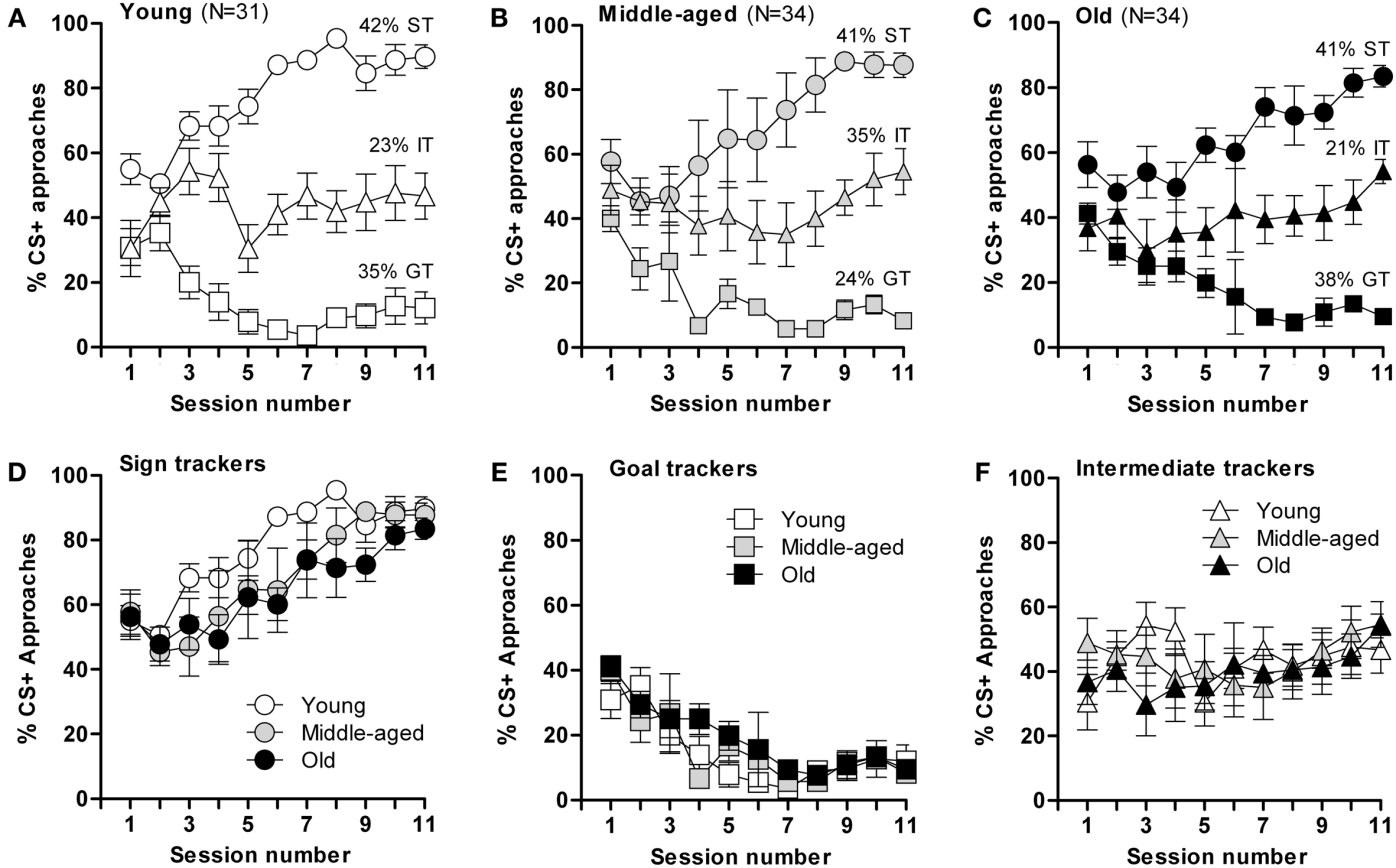
**Acquisition of conditioned responses during autoshaping**. Mice displayed different conditioned responses, designated as sign-tracking (ST, predominantly approached the CS+), goal-tracking (GT, predominantly approached the US) and intermediate-tracking (IT, alternated between CS+ and US with approximately equal frequency) behaviors. Autoshaping was monitored over 11 sessions; in each session, mice received 15 CS+ and 15 CS− presentations. **(A)** The young mice (*n* = 31) segregated into sign-trackers (ST; *n* = 13), goal-trackers (GT; *n* = 11) and intermediate trackers (IT; *n* = 7). **(B)** The middle-aged mice (*n* = 34) segregated into sign-trackers (ST; *n* = 14), goal-trackers (GT; *n* = 8) and intermediate trackers (IT; *n* = 12). **(C)** The old mice (*n* = 34) segregated into sign-trackers (ST; *n* = 14), goal-trackers (GT; *n* = 13) and intermediate trackers (IT; *n* = 7). **(D–F)** CS+ approaches by, respectively, sign−, goal− and intermediate-tracking mice of different ages. Data are presented as means ± s.e.m.

Based on their CR on the last 3 days of the training schedule, mice were categorized as sign trackers (ST, at least 70% of approaches to CS+), goal trackers (GT, <20% CS+ approaches), or intermediate trackers (IT, 20–70% CS+ approaches). Interestingly, segregation into ST, GT, and IT was similarly distributed across all three age groups; young: 42% of mice were ST, 35% GT, and 23% IT [*F*_(2,303)_ = 409.8; *p* ≤ 0.0001]; middle-aged: 41% of mice were ST, 24% GT, and 35% IT [*F*_(2,316)_ = 78.9; *p* ≤ 0.0001]; old: 41% ST, 38% GT, and 21% IT [*F*_(2,328)_ = 133; *p* ≤ 0.0001] (Figures 2A–C), but the rate of task acquisition did not differ between age groups (Figures 2D–F). Notably, while each of the three age groups had significantly different body masses, this parameter did not differ between mice displaying ST, GT, or IT behavior within each age group (data not shown). Briefly, the results of this test demonstrate that appetitive learning abilities are robustly conserved during aging and, that ST, GT, and IT behaviors are innate characteristics that do not shift with age.

### Motivation for appetitive reward is intact in aging mice

**Figure 3:**
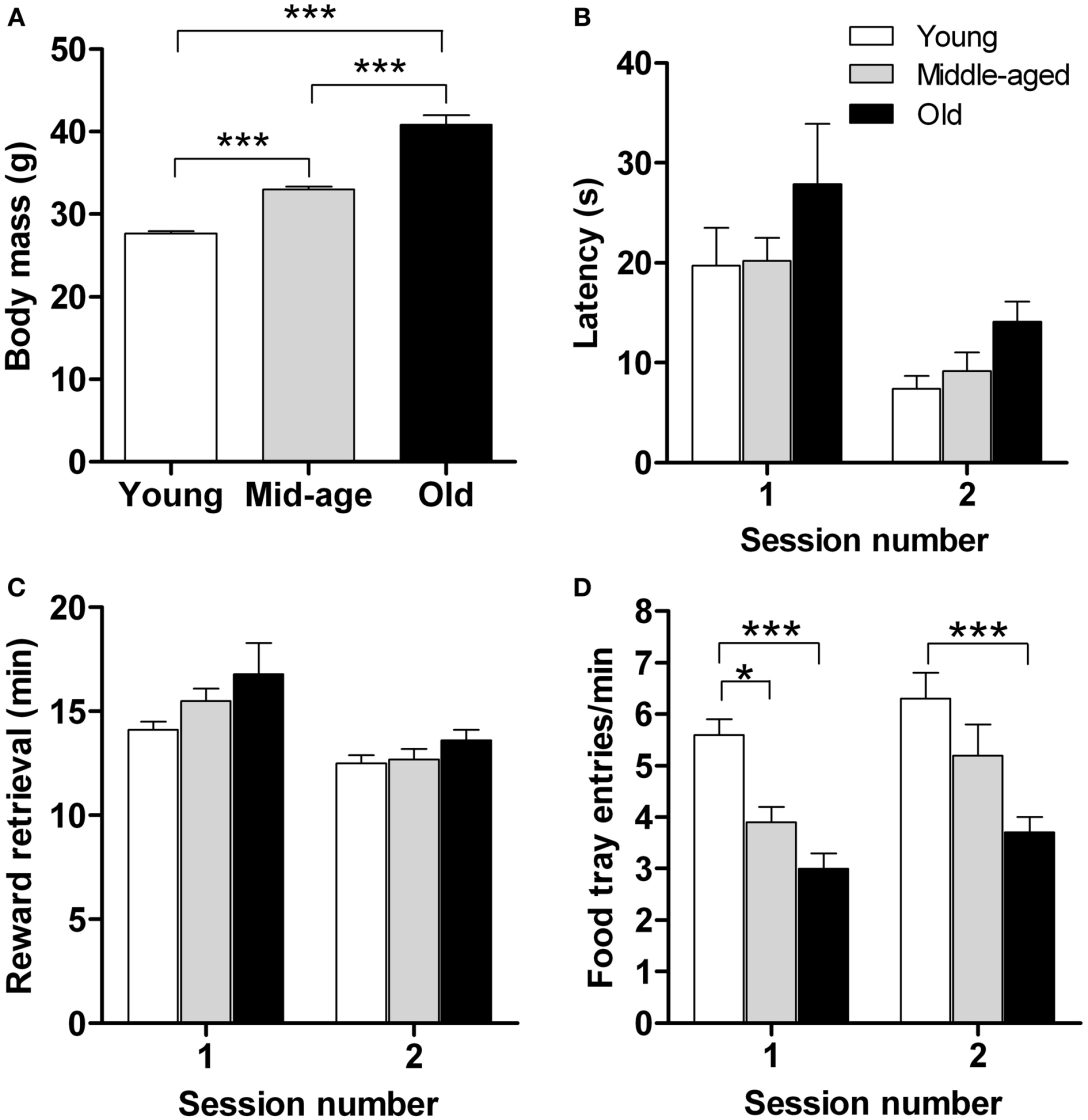
**Motivation for food reward does not differ between young (*n* = 15), middle-aged (*n* = 16), and old (*n* = 14) mice**. Animals were rewarded with sweetened milk which is more rewarding than their standard food pellets. For each age group, initial body masses **(A)**, mean latency to approach the reward **(B)**, time taken to retrieve (and consume) the food reward **(C)**, and the number of food tray entries **(D)** are shown. Measurements were made over 2 sessions, with 15 reward deliveries in each. Results shown represent means ± s.e.m. ^*^ and ^***^ denote significant differences (*P* < 0.05 and *P* < 0.001, respectively) between the indicated pairs of data.

Motivation is a key factor in reward learning (Dayan and Balleine, 2002) and eating (Kringelbach et al., 2012) behavior. Although the previous set of data showed that the capacity for implicit learning remains intact in aged mice, we consider of interest to examine whether aging alters motivation for rewarding foods. To this end we monitored latency to approach, retrieve and consume sweetened milk (reward) and the number of food-tray entries in a food retrieval test, *independent of learning strategies*. The test was performed during a period of caloric restriction in young (*n* = 15), middle-aged (*n* = 16), and old (*n* = 15) mice; between-group starting body masses were significantly different (Figure 3A; *p* < 0.001).

As shown in Figure 3B, there were no significant age-related differences in latency of first approach-to-reward. Notably, however, approach latency decreased significantly, and in an “age-independent manner,” in the second test session, indicating familiarity with the task and that it had been learnt [*F*_(1,83)_ = 23.8; *p* ≤ 0.0001]. Also, all age groups retrieved and consumed the sweetened milk reward within comparable times, their performance being significantly enhanced in the second test session [*F*_(1,83)_ = 19.3; *p* ≤ 0.0001] (Figure 3C). Significant between-age group differences were observed in the number of food-tray entries [*F*_(2,83)_ = 19.7; *p* ≤ 0.0001] (Figure 3D): young mice entered the food-tray more frequently than old mice on both test days (*p* < 0.001) and more frequently than the middle-aged group on the first day of testing (*p* < 0.05), consistent with the greater exploratory activity generally observed when younger mice are placed in novel environments (Fahlström et al., 2012). Analysis of the various parameters used to assess motivation to retrieve and consume a food reward failed to reveal a significant effect of either age or type of conditioned response (cf. Figure 2). Overall, the results of this experiment show that motivation for rewarding foods is not altered by aging.

### Hedonic preferences endure even in old age

**Figure 4:**
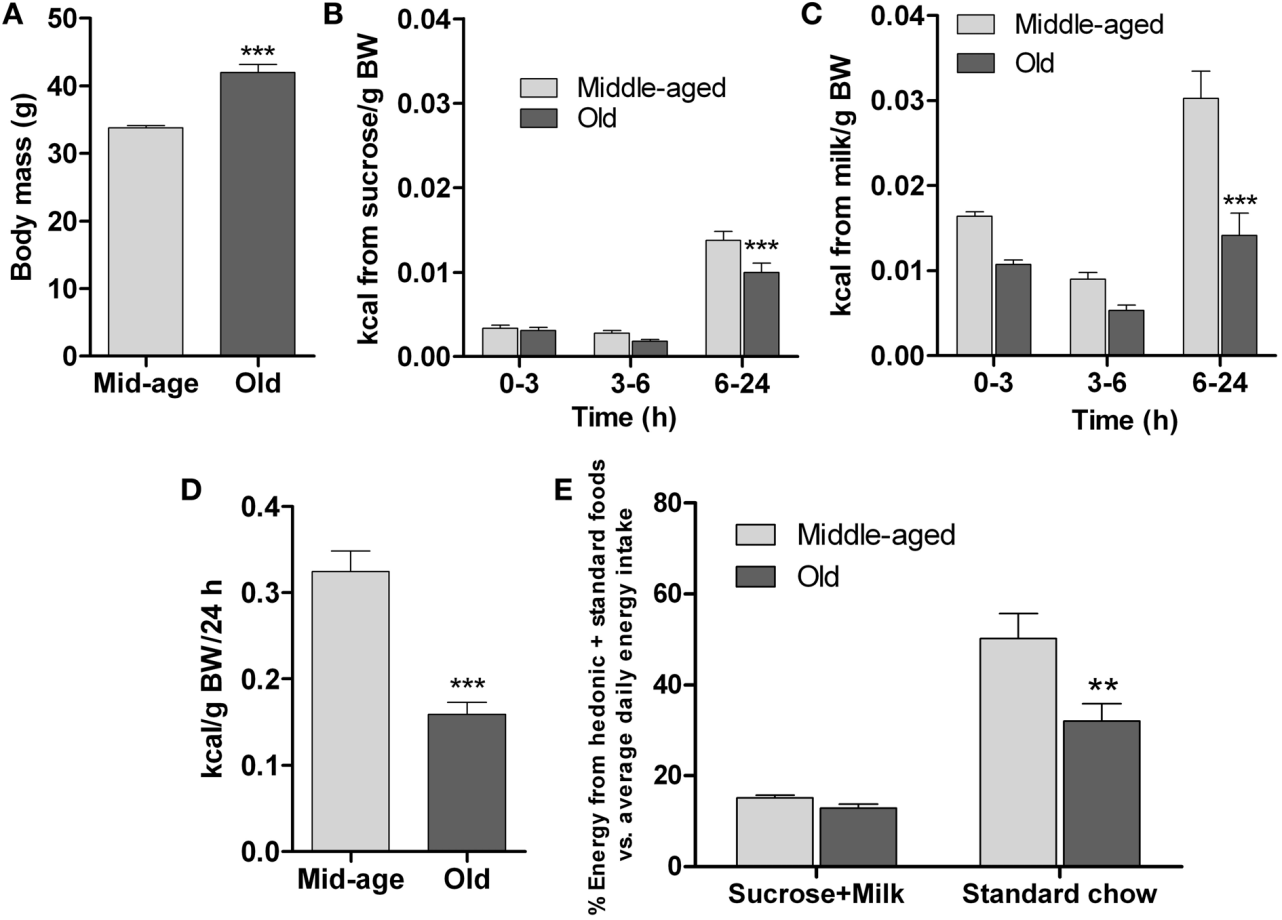
**Hedonic preference test**. The test compared consumption of two isocaloric palatable liquid-foods sucrose (15%) and milk (5% fat) in presence of unlimited amounts of standard chow diet by middle-aged (6 months, *n* = 20) and old (13 months, *n* = 15) mice. **(A)** Initial body mass. **(B–C)** Sucrose and milk consumption during different intervals over 24 h. **(D)** Total energy intake (hedonic foods+standard chow diet) in 24 h, expressed relative to body mass. **(E)** Relative (%) energy derived from either hedonic liquid foods (sucrose or milk) or standard chow during a 24 h test period vs. average daily energy derived from standard chow food during pre-test monitoring. All data are shown as means ± s.e.m. ^**^ and ^***^ denote significant differences (*P* < 0.01 and *P* < 0.001, respectively) between middle-aged and old mice.

Since food preference is an important factor in the development of eating patterns (Berridge and Kringelbach, 2013), examination of this parameter in differently-aged mice was undertaken to complement the data reported above. This was achieved by comparing the consumption of highly-rewarding (sweet and fatty), isocaloric liquid foods vs. standard solid chow over 24 h; animals previously had ad lib access to standard diet. The study was done in middle-aged and old mice, whose body weights were significantly different (*p* < 0.001, Figure 4A).

The data in Figures 4B,C depict the temporal patterns of consumption of sucrose and milk in the different age groups. Over 24 h, both groups consumed volumes of the liquid diets that exceeded their usual daily consumption of water [middle-aged: average water consumption = 2.7 ml/24 h; sucrose consumed = 10.5 ml/24, (*p* < 0.001) (vs. water); milk consumed = 29.4 ml/24, (*p* < 0.001) (vs. water); old: average water consumption = 2.3 ml/24 h; sucrose consumed = 9.75 ml/24, (*p* < 0.001) (vs. water); milk consumed = 19.5 ml/24, (*p* < 0.001) (vs. water); see Supplementary Figure 1 for water consumption data]. Further, as shown in Figures 4B,C, as compared to their middle-aged counterparts, the old mice consumed significantly less sucrose [*F*_(1,99)_ = 9; *p* = 0.003] and milk [*F*_(1,99)_ = 31.5; *p* ≤ 0.0001] between 6 and 24 h, although they showed similar rates of consumption during the first 6 h of testing; within group comparisons revealed that mice of both ages preferred milk over sucrose (*p* < 0.001). Since the two liquid diets were isocaloric, these findings indicate a role of hedonic factors (smell, taste, texture) in the regulation of preference.

Computation of the total energy (calories/kg BW) derived from the combination of the sucrose and milk solutions and standard diet during the 24 h exposure to the food choice paradigm revealed that the energy intake of middle-aged mice was significantly higher than that of the old mice (*p* < 0.001; Figure 4D). Interestingly, the relative amounts of energy derived from the highly palatable liquid diets did not differ between the two age groups (Figure 4E); however, the younger mice derived relatively more energy from the standard diet (*p* < 0.05; Figure 4E), consistent with their higher total energy intake.

In brief, these findings indicate that (i) hedonic set-points are not changed during aging in the mouse, and (ii) that old mice can match their total daily calorific intake, derived from hedonically-loaded foods and a standard chow diet, to their (reduced) metabolic requirements just as efficiently as middle-aged animals.

## Discussion

The main finding of our experiments is that three essential components of feeding behavior—conditioned learning, motivation and, ability to choose foods based on their hedonic properties—remain intact during aging in the mouse. This evidence is important in light of the growing use of rodent models for understanding the mechanisms underlying human obesity and in particular, the search for the cause of mid-life obesity; the latter associates strongly with a spectrum of metabolic, neurological and psychiatric diseases during human aging.

Although there is a large literature on age-induced impairments of declarative (explicit) learning and memory and on the processes and mechanisms responsible for such impairments (Hedden and Gabrieli, 2004; Driscoll and Sutherland, 2005), little is known about how aging affects implicit memory. Implicit memory plays an important role in feeding behavior, exemplified in the work by Schoenbaum et al. (2002); Roman et al. (1996); Renteria et al. (2008); these authors reported that the acquisition and recall of conditioned responses (e.g., flavor-reward and odor-reward associations) are negatively influenced by aging in rats. In contrast, we found that young, middle-aged and old mice do not differ in their performance in pavlovian (autoshaping) and operant conditioning paradigms, involving passive and active associative learning. Both, young mice (Harb and Almeida, 2014) and rats (Lomanowska et al., 2011; Anderson et al., 2013), develop sign−, goal− or intermediate-tracking behaviors during pavlovian conditioning. Similar conditioned learning responses were observed in the present study and, importantly, the distribution of response patterns was not a function of age, indicating that all the known features of conditioned learning are preserved during aging.

The results from the two appetitive associative learning paradigms used in the present work are particularly relevant to the study of eating behavior in the context of overweight and obesity. External cues have an important influence over the amount of food consumed by hungry and satiated rodents (Weingarten, 1983; Petrovich et al., 2007). Appetitive conditioning has also been implicated in human feeding behavior; for example, cues provided in the media and the general obesogenic environment that pervades modern societies strongly influence eating choices and eating patterns in children (Jansen et al., 2003; Halford et al., 2008; Birch and Anzman-Frasca, 2011), adolescents (Scully et al., 2012), and adults (Scully et al., 2009), independently of body mass status (Ferriday and Brunstrom, 2011; Ziauddeen et al., 2012). The present findings demonstrate that subjects of any age can attribute incentive salience to food cues, thus placing them at risk for maladaptive behaviors, e.g., overeating in absence of metabolic need. However, we previously found that sign-tracking conditioned responding—a possible surrogate of compulsive behavior—does not predict propensity to overeat or develop obesity in the mouse (Harb and Almeida, 2014).

Besides the role of external cues in the conditioning of feeding behavior, the inherent sensory (odor, visual appearance, taste and texture) and physical (energy content) properties of foods provide the motivational drive to seek food and to determine the amount of food ingested (Mehiel and Bolles, 1988; Rolls, 2010; Beeler et al., 2012; Fernstrom et al., 2012; Desmarchelier et al., 2013; Li et al., 2013). Since sensory processing is altered in many older humans, a phenomenon best exemplified by the so-called “anorexia of aging” (Morley, 1997; Donini et al., 2003; de Boer et al., 2013), we compared the motivation to eat, including the consumption of foods with high hedonic value, in young, middle-aged and old mice. An experiment in which we measured the time taken to retrieve and consume a rewarding food revealed that although younger mice are more reactive than their older counterparts in terms of stimulus touches, motivation to eat remains stable throughout the lifespan of the mouse. This finding contrasts with that of an earlier report that older rats have reduced motivational drive (Frutos et al., 2012); however, the results of that study, obtained in the “incentive runway” test, may be more reflective of differences in locomotor speed (older rats were slower) and body mass and energy reserves (greater in aged animals) than of motivational drive *per se*.

Given that the capacity for associative learning and motivation to eat do not differ between mice of different ages and, that responses to the hedonic qualities of foods may be impaired in aging humans (Morley, 1997), we examined whether the display of hedonic preference for palatable foods is altered in aging mice in this study. Our experimental results show that young and old mice do not differ in their consumption of milk or sucrose during the first 6 h of parallel presentation of these hedonically-charged foods with standard chow diet. On the other hand, while the relative consumption rates of milk and sucrose did not differ between young and old mice over the 24 h of testing, the older animals consumed less of their standard diet. These observations show that aging does not interfere with hedonic choice-making and, that aged mice retain their ability to adjust calorific intake to match their daily energy requirements; the lower caloric intake by older animals may be attributed to the fact that, as compared to young mice, they are less active (Ingram, 2000) and thus expend lower amounts of energy; at the same time, older mice have access to a larger energy depot (fat mass) as compared to their younger conspecifics (Hariri and Thibault, 2010). Overall, our findings are consistent with those of Frutos et al. (2012) who also reported that young and old rats do not differ in their hedonic reactions to sucrose and corn oil and that older rats show more rapid signs of satiation.

The key findings of the present study are as follows: firstly, we show that implicit (associative) memory for food cues remains intact in old mice. This is not surprising since feeding provides essential nutrients and is the main source of energy for all living organisms. However, given the predominant focus of research on the causes and mechanisms of age-related decline in explicit memory, the present results raise awareness of the need for exploration of whether other types of implicit memory might also be preserved during aging. Secondly, our experiments indicate that aging does not necessarily reduce proneness to maladaptive feeding behavior; this is because motivation and responses to hedonic signals about food are retained throughout mouse aging. Interestingly, however, hypofunction of the neuroanatomical and neurochemical substrates that mediate motivation and reward has been reported in aged humans that have high amounts of abdominal fat and/or a high body mass index (Green et al., 2011). Thirdly, in contrast to humans who are at risk of developing obesity because hedonic signals prevail over homeostatic control mechanisms, mice are apparently able to maintain a balance between energetic demand and conflicting hedonic stimuli. Although a highly-conserved behavior, eating is a complex behavior whose regulation and execution represents integration of energetic, metabolic, sensory, cognitive, and motor functions; further, implicit memory plays an important role in eating. Age-related impairments in explicit memory are well established; the results presented in this study show, for the first time to the best of our knowledge, that implicit memory is unaltered in ageing. Thus, the risk for older humans to be conditioned by food-related hedonic cues is likely to be greater than previously thought; this combined with age-related reductions in physical activity, also place them at risk for developing obesity and its associated disorders.

## Author contributions

Mazen R. Harb and Osborne F. X. Almeida designed the study. Mazen R. Harb performed the experiments and collected and analyzed the data. Nuno Sousa and Joseph Zihl made critical comments on the manuscript. Mazen R. Harb and Osborne F. X. Almeida wrote the paper. All authors read and approved the final manuscript.

### Conflict of interest statement

The authors declare that the research was conducted in the absence of any commercial or financial relationships that could be construed as a potential conflict of interest.
