# Supplementary figures and images for: Reward components of feeding behavior are preserved during mouse aging
Source: Front Aging Neurosci. 2014 Sep 16;6:242. doi: 10.3389/fnagi.2014.00242 (PMC4165288; doi:10.3389/fnagi.2014.00242)

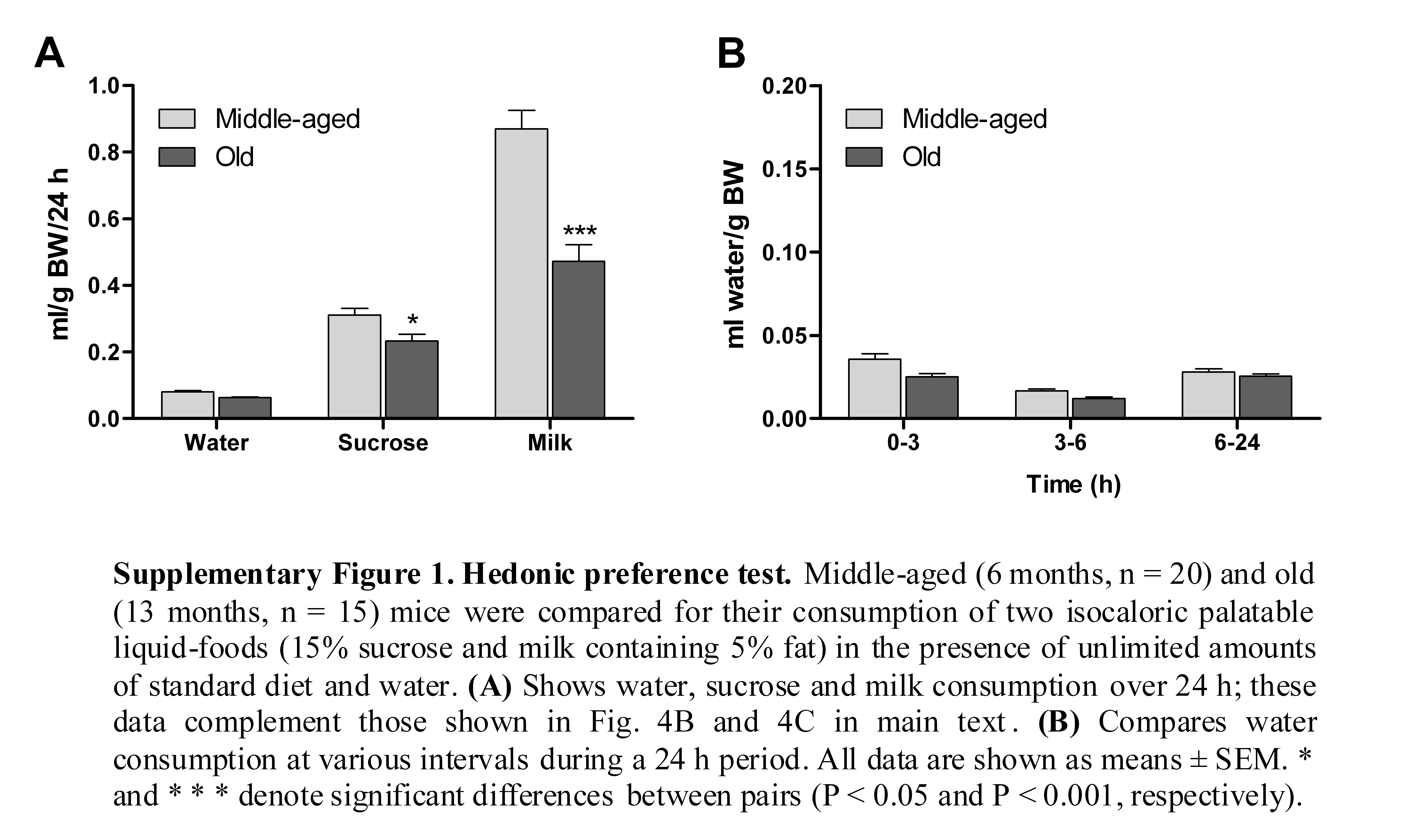

Supplement: Supplementary file 1 [file Image1.JPEG]
